# Cost Analysis of Pelvic Exenteration Surgery for Advanced Pelvic Malignancy

**DOI:** 10.1245/s10434-024-16227-3

**Published:** 2024-09-16

**Authors:** Charles W. G. Risbey, Kilian G. M. Brown, Michael Solomon, Kate McBride, Daniel Steffens

**Affiliations:** 1 Surgical Outcomes Research Centre (SOuRCe), Royal Prince Alfred Hospital, Camperdown, NSW Australia; 2 Faculty of Medicine and Health, Central Clinical School, The University of Sydney, Sydney, Australia; 3 Department of Colorectal Surgery, Royal Prince Alfred Hospital, Sydney, Australia; 4 RPA Institute of Academic Surgery, Royal Prince Alfred Hospital, Sydney, Australia

**Keywords:** Pelvic exenteration, Locally advanced rectal cancer, Locally advanced pelvic malignancy, Health economics, Cost analysis

## Abstract

**Background:**

Pelvic exenteration (PE) is a radical procedure involving multi-visceral resection for locally advanced pelvic malignancies. Such radical surgery is associated with prolonged operating theater time and hospital stay, as well as a substantial risk of postoperative complications, and therefore significant financial cost. This study aimed to comprehensively detail the inpatient cost of PE at a specialist center in the Australian public sector.

**Methods:**

A retrospective costing review of consecutive PE operations at Royal Prince Alfred Hospital in Sydney between March 2014 and June 2022 was performed. Clinical data were extracted from a prospectively maintained database, and in-hospital costing data were provided by the hospital Performance Unit. All statistical analyses were performed using SPSS.

**Results:**

Pelvic exenteration was performed for 461 patients, of whom 283 (61 %) had primary or recurrent rectal cancer, 160 (35 %) had primary or recurrent non-rectal cancer, and 18 (4 %) had a benign indication. The median admission cost was $108,259.4 ($86,620.8–$144,429.3) (Australian dollars [AUD]), with the highest costs for staffing followed by the operating room. Overall, admission costs were higher for complete PE (*p* < 0.001), PE combined with cytoreductive surgery (CRS) (*p* < 0.001), and older patients (*p* = 0.006).

**Discussion:**

The total admission cost for patients undergoing PE reflects the complexity of the procedure and the multidisciplinary requirement. Patients of advanced age undergoing complete PE and PE combined with CRS incurred greater costs, but the requirement of a sacrectomy, vertical rectus abdominal flap reconstruction, major nerve or vascular excision, or repair were not associated with higher overall cost in the multivariate analysis.

**Supplementary Information:**

The online version contains supplementary material available at 10.1245/s10434-024-16227-3.

Pelvic exenteration (PE), a major surgical procedure, was first developed in the mid-20th century as a palliative operation for locally advanced, recurrent cervical carcinoma.^1^ With advances in oncologic resection, PE has evolved to be the standard of care for selected patients with locally advanced pelvic malignancy, and occasionally can be considered for benign pathology such as chronic fistulating pelvic sepsis.^1–3^

The PE procedure involves en bloc resection of multiple pelvic viscera with any involved contiguous pelvic soft tissue or bony structures. As surgical techniques have been refined, increasingly radical exenterative resections have been performed at specialized units in pursuit of clear surgical margins (the most important indicator for long-term disease-free survival) in patients who have historically been considered incurable.^1,4^

Several subspecialist surgical teams often are involved with PE surgeries. Many patients require complex urologic, vascular, orthopedic, or soft tissue reconstruction, contributing to substantial operating time, postoperative care requirements, and need for rehabilitation.^5^ Such radical surgery also can be associated with significant postoperative morbidity, with some patients requiring prolonged postoperative inpatient hospital admissions and the involvement of multiple specialist medical and allied health teams before discharge from acute hospital care.^5,6^ Given that prolonged operative times are expected, that extensive multidisciplinary input is required, and that a challenging postoperative hospital admission often occurs, the economic costs of providing such a complex surgical service are significant.^1^

Previous work from the authors' unit found the median total inpatient cost of patients undergoing PE to be $56,638 (Australian dollars [AUD]). However, these data are now more than a decade old and, considering the medical and surgical advances in the area, may no longer be representative of the true cost.^7^ Specifically, in Australia, public funds are allocated through activity-based funding (ABF) systems, which use diagnosis-related groups (DRGs), meaning an accurate understanding of the costs incurred by PE are required for appropriate funding of the units providing the service. As such, given the increasing costs of health care in Australia as well as increasing interest and advances in exenteration surgery, realistic and contemporary expectations for funding of such low-volume, high-cost surgery are relevant for both existing exenteration centers and prospective programs.^8^

Overall, a better of understanding the financial implications of PE will facilitate improved resource allocation and health economic planning at a population level. Indeed, both individual PE units and broader health care networks stand to benefit from increased understanding of the financial implications of running a specialist PE service. As such, the primary aim of this study was to comprehensively describe the total inpatient hospital costs associated with PE at a high-volume specialist exenteration service.

## Methods

This study retrospectively analyzed the cost for consecutive patients undergoing PE at Royal Prince Alfred Hospital (RPA) in Sydney, Australia. The study included patients age 18 years or older undergoing PE for any indication, benign or malignant, from March 2014 through June 2022. Patients undergoing either partial or complete PE were included, with partial PE defined as the en bloc resection of a minimum of three pelvic compartments and/or bone or lateral neurovascular resection, and complete PE as the resection of all pelvic compartments and viscera.^9^

The indications for PE were stratified into the following five cohorts: primary rectal cancer, recurrent rectal cancer, other primary cancer, other recurrent cancer, and non-cancerous etiologies.^5^ “Other” cancers represent a heterogeneous group, including genitourinary, anal, soft tissue, and bone tumors.

Patients meeting the inclusion criteria for this study were identified from a prospectively maintained institutional PE database.^5,9^ Patient demographic, tumor, and surgical data were extracted directly from the database. Place of residence was defined as per the Bureau of Statistics (ABS) Australian Statistical Geography Standard (ASGS) Edition 3 Remoteness Structure, with patients further stratified to reflect interstate or overseas origin.^10^ Tumor margins were defined according to the R classification as follows: R0 (microscopically clear surgical margins, with only normal tissue present at the margin), R1 (residual microscopic disease at the tumor margin, and R2 (residual macroscopic disease at the tumor margin).^9^

Detailed cost data were obtained from the RPA performance unit using the same top-down approach as previously described by our group.^11–13^ Specifically, individual costs for each patient admission were extracted from the annual inpatient fractions (iFRACs) costing review. Spending by each cost center within the hospital was captured using this methodology and reflects the total costs incurred by the health district for each case, both clinical and administrative. These values reflect the total cost per patient associated with providing a high-volume, complex exenteration service. The pricing framework is set by the Independent Health and Aged Care Pricing Authority (IHACPA), which underpins the nationally consistent ABF system for public hospitals in Australia.

Individual expenses were categorized into cost groups, which were then arranged into the six cost buckets including staff, critical care, diagnostic, operating room, ward, and other costs to improve interpretation and clinical relevance, as previously described.^11,12^ Costs classified as “other costs” included non-clinical administrative costs as well as food and domestic services, patient transport, and accounting costs associated with the hospital (Table S1). To account for health-adjusted inflation, all reported costs have been standardized to 2022 $AUD prices using Australian Institute of Health and Welfare (AIHW) annual health inflation rates, which averaged 2.04 % during the decade preceding 2022.^8,14^ To the authors’ knowledge, major changes occurred within our health district or national health care system more broadly that may have influenced the overall health care cost.

Statistical analyses were completed using SPSS version 29 (IBM, Armonk, NY, USA) and R Statistical Software version 4.3.0 (R Foundation for Statistical Computing, Vienna, Austria). Continuous variables were assessed for normality using the Shapiro-Wilk test. Statistical significance was assessed using either the Mann-Whitney *U* or Kruskal Wallis test for continuous, non-parametric variables. All tests of significance were two-tailed, and *p* values lower than 0.05 were considered significant. A multivariate regression analysis with backward variable selection incorporating variables with significant univariate association (*p* < 0.2) also was performed. Descriptive statistics were reported as median (IQR) for continuous variables and as frequencies (%) for categorical variables. All costs are expressed in Australian dollars (AUD) unless otherwise stated.

This observational cohort study was conducted in accordance with the Strengthening the Reporting of Observational Studies in Epidemiology (STROBE) statement.^15^

## Results

### Baseline Demographic and Surgical Characteristics

This study included 461 patients. The median age was 62 years, with an interquartile range (IQR) of 51 to 69 years, and 218 (47 %) patients were female (Table 1). The PE procedure was used to treat 144 (31 %) patients with primary rectal malignancy, 139 (30 %) patients with recurrent rectal malignancy, 63 (14 %) patients with primary non-rectal malignancy, 97 (21 %) patients with non-rectal malignancy, and 18 (4 %) patients with a benign indication. The majority of the patients were from New South Wales (NSW). However, 93 (20 %) patients traveled from interstate, and 2 patients (0.4 %) were international.

**Table 1:** Characteristics of the study population

Characteristics	Primary rectal (*n* = 144)	Recurrent rectal (*n* = 139)	Primary other (*n* = 63)	Recurrent other (*n* = 97)	Non-cancer (*n* = 18)	Total (*n* = 461)
Age (years)	60 (50–70	63 (54–70)	58 (46–68)	59 (46–69)	60.5 (56–71)	62 (51–69)
Sex (female)	56 (38.9)	51 (36.7)	40 (63.5)	62 (63.9)	9 (50.0)	218 (47.3)
Place of residence						
NSW metro	48 (33.3)	65 (46.8)	33 (52.4)	47 (48.5)	11 61.1)	204 (44.3)
NSW regional or remote	67 (46.5)	40 (28.8)	20 (31.7)	29 (29.9)	6 (33.3)	162 (35.1)
Interstate	28 (19.4)	33 (23.7)	10 (15.9)	21 (21.6)	1 (5.6)	93 (20.2)
Overseas	1 (0.7)	1 (0.7)	0 (0.0)	0 (0.0)	0 (0.0)	2 (0.4)
Type						
Complete	79 (54.9)	73 (52.5)	27 (42.9)	59 (60.8)	6 (33.3)	244 (52.9)
Partial	65 (45.1)	66 (47.5)	36 (57.1)	38 (39.2)	12 (66.7)	217 (47.1)
Neoadjuvant therapy						
Chemotherapy	125 (86.8)	59 (42.8)	15 (23.8)	26 (26.8)	1 (5.6)	226 (49.1)
Radiotherapy	126 (87.5)	53 (38.4)	14 (22.2)	14 (14.6)	0 (0.0)	207 (45.1)
Curative intent	140 (97.9)	138 (99.3)	59 (93.7)	84 (86.6)	18 (100)	439 (95.4)
Combined CRS +/- HIPEC	2 (1.4)	3 (2.2)	3 (4.8)	6 (6.2)	0 (0)	14 (3.0)
R margin						
R0	130 (90.3)	110 (79.1)	49 (77.8)	72 (74.2)	8 (44.4)	369 (80.0)
R1/2	14 (9.7)	25 (18.0)	14 (22.2)	23 (23.7)	1 (5.6)	77 (16.7)
Not assessable	0 (0.0)	4 (2.9)	0 (0.0)	2 (2.1)	9 (50.0)	15 (3.3)
Operating Time (hours)	9.1 (7.4–10.9)	10.1 (8.1–11.9)	8.8 (6.8–11.3)	10.0 (8.9–12.1)	8.8 (5.1–10.4)	9.5 (7.9–11.4)
Length of ICU stay (days)	3.0 (2.0–5.0)	4.0 (2.0–6.0)	3.0 (2.0–5.0)	4.0 (3.0–6.0)	3.5 (2.0–5.7)	4.0 (2.0–5.0)
Length of hospital stay (days)	19.0 (14.3–29.0)	23.0 (16.0–33.0)	19.0 (14.0–27.0)	23.0 (16.0–39.0)	23.5 (16.8–30.3)	22.0 (15.0–31.0)

Data presented as median (IQR) or n (%).

*NSW* New South Wales; *CRS* Cytoreductive surgery; *HIPEC* Hyperthermic intraperitoneal chemotherapy

Complete PE was performed for 244 (53 %) patients, with the remaining 217 patients (47 %) undergoing partial PE (Table 1). Neoadjuvant chemotherapy was completed by 226 (49 %) patients, and neoadjuvant radiotherapy by 207 (45 %) patients. The vast majority of the patients (95 %) underwent PE with curative intent. In 369 (80 %) cases, R0 tumor margins were achieved. For 14 (3 %) patients, a combination of PE and cytoreductive surgery (CRS) with or without heated intraperitoneal chemotherapy (HIPEC) was used.

The median operative time was 9.5 h, and 92 (20 %) procedures required more than 12 h to complete. The median intensive care unit (ICU) length of stay (LOS) was 4 days (range, 0–71 days), and the median total hospital admission was 22 days (range, 2–185 days), both demonstrating positively skewed distributions (Table 1). One or more postoperative complications were encountered by 418 (91 %) of the patients, with 82 (18 %) patients requiring an unplanned return to the operating theater during the postoperative period.

### Total Admission Cost

**Fig. 1:**
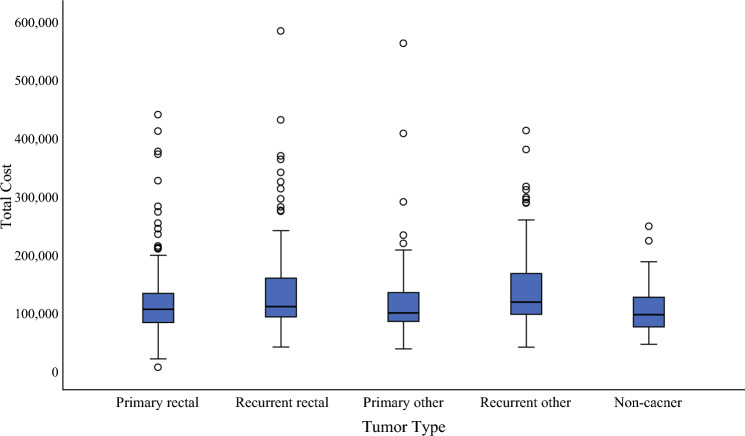
Median total cost by tumour type (2022 $AUD)

Overall, the median total inpatient cost for PE was $108,259.4 AUD (IQR, $86,620.8–144,429.3 AUD, standardized to 2022 AUD$ to account for health care-adjusted inflation. The median cost for PE based on surgical indication ranged from $95,858.9 AUD for benign indications to $118,129.6 AUD for recurrent malignancy of non-rectal origin (Fig. 1; Table 2). Overall, the cost for PE was positively skewed, with 34 (7 %) patients exceeding $250,000 AUD in total admission costs and 2 (0.4 %) patients exceeding $500,000 AUD in total admission costs.

**Table 2:** Detailed cost breakdown according to type of pelvic exenteration surgery

Variable	Staff	Critical care	Diagnostic	Theatre	Ward	Other	Total
Tumour type							
Primary rectal	35,927.0 (29,766.9–46,014.1)	8,642.4 (4,706.2–14,238.2)	8,124.2 (4,826.1–15,390.4)	24,421.5 (18,028.0–30,369.0)	5,023.7 (3,910.6–7,316.7)	17,679.4 (14,210.3–26,114.8)	104,541.7 (82,867.8–132,984.7)
Recurrent rectal	38,148.5 (32,928.3–47,880.2)	10,165.5 (6,540.6–17,384.7)	8,359.6 (4,266.5–16,123.8)	26,683.5 (19,897.3–34,775.0)	5,460.1 (4,409.1–7,253.8)	20,346.0 (16,283.8–32,727.3)	109,690.6 (91,598.9–160,068.4)
Primary other	36,707.5 (30,131.8–45,080.3)	8,566.5 (5,292.7–13,032.6)	8,159.0 (3,925.7–15,999.9)	22,648.8 (15,557.4–31,661.0)	5,207.1 (4,241.8–6,563.9)	18,069.3 (13,247.2–25,335.9)	98,363.3 (83,175.7–136,274.1)
Recurrent other	40,090.8 (32,094.8–56,358.3)	11,271.8 (6,843.7–19,237.1)	11,097.9 (4,825.4–19,254.0)	26,530.1 (19,692.4–35,238.2)	5,730.4 (4,639.3–9,085.4)	22,280.5 (16,735.4–33,032.6)	118,129.6 (96,503.0–171,822.1)
Non-cancer	38,040.2 (27,060.9–46,281.5)	7,941.9 (5,494.8–17,330.1)	10,051.1 (3,226.7–16,199.4)	20,115.0 (13,019.7–26,503.2)	5,589.7 (3,165.5–8,366.1)	17,671.1 (12,434.7–23,778.0)	95,858.9 (73,576.7–126,643.0)
PE type							
Complete	40,182.7 (34,364.3–53,651.4)	11,008.5 (6,734.2–17,055.4)	11,814.9 (6,422.3–19,272.7)	28,244.4 (22,997.4–37,435.6)	5,916.7 (4,896.8–8,454.0)	22,735.1 (17,764.0–32,794.3)	121,469.8 (101,789.6–163,491.9)
Partial	34,365.1 (28,434.7–42,641.6)	8,414.9 (5,224.0–14,279.8)	5,639.7 (3,112.6–11,185.1)	19,666.7 (14,009.3–28,399.4)	4,547.2 (3,764.8–6,412.6)	15,609.6 (11,325.4–23,702.3)	92,771.8 (71,906.6–122,611.0)
All	37,479.5 (31,012.7–47,385.6)	9,563.4 (5,783.0–16,220.2)	8,798.5 (4,370.9–16,424.4)	24,982.4 (18,236.6–32,664.2)	5,319.4 (4,211.7–7,495.1)	19,964.1 (14,460.7–27,907.7)	108,259.4 (86,620.8–144,429.3)

Data presented as median (IQR); All costs are 2022 $AUD

The proportion of total cost accounted for by individual cost buckets is presented in Table 2. Staffing costs, comprising the total expenses incurred by allied health and medical and nursing staff, represented the highest proportion of the total cost per patient (35 %, median staffing expenses $37,479.5 AUD per patient). The costs associated with medical staff were the primary driver of these costs, with a median of $19,011.1 AUD per patient directly attributable to medical staff. Nursing and allied health expenses were relatively lower, with attributable costs of $12,119.6 and $5,973.0 AUD, respectively.

**Fig. 2:**
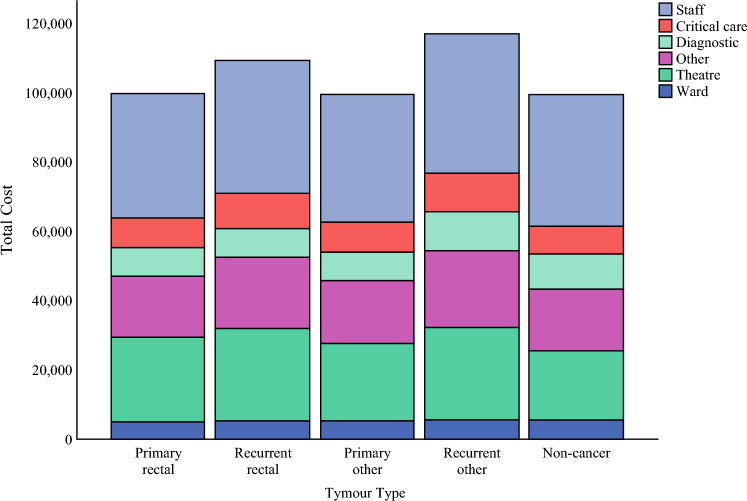
Cost breakdown by tumour type (2022 $AUD)

Operating theater costs and “other costs” were the next most substantial cost buckets, with median values of $24,982.4 and $19,964.1 AUD respectively per case. Critical care, diagnostic, and ward costs made up the remainder of the total expenditure, with each cost bucket amounting to less than $10,000 AUD per case. Figure 2 illustrates the cost breakdown by cost buckets for different tumor types.

### Factors Impacting Overall Admission Cost

Univariate analysis demonstrated that the inpatient cost for PE did not differ between rectal and non-rectal malignancy (*p* = 0.500) or for increasing age (*p* = 0.065). However, the median total cost was higher for men than for women ($113,808.2 vs $103,704.1 AUD; *p* = 0.005), for complete PE than for partial PE ($121,469.8 vs $92,771.8 AUD; *p* < 0.001), for PE treatment of recurrent than of primary malignancy ($114,723.9 vs $103,433.6 AUD; *p* = 0.002), for PE with than without CRS ± HIPEC ($195,521.5 vs $107,608.1 AUD; *p* < 0.001), and for PE with palliative than with curative intent ($142,788.9 vs $107,777.1 AUD; *p* = 0.019) based on univariate analysis (Table 3).

**Table 3:** Effect of surgical characteristics on total cost

	Univariate analysis			Multivariate analysis	
Variable	n	Median cost (IQR)	*p* value	Coefficient (Std. error)	*p* value
Year of surgery (years)			0.548		
2014–16	53	104,139.6 (73,448.4–141,168.4)			
2017–19	224	110,452.6 (85,103.9–159,866.2)			
2020–2022	184	108,419.3 (90,704.7–136,875.6)			
Age (years)			0.065	770.4 (276.9) ^d^	0.006
20–39	36	95,728.2 (66,500.8–141,922.8)			
40–59	176	103,534.5 (83,551.6–139,355.2)			
60–79	234	111,528.9 (91,369.4–154,667.9)			
80–99	15	115,050.6 (94,781.1–163,744.3)			
Sex			0.005		
Female	218	103,704.1 (83,302.1–133,146.9)			
Male	243	113,808.2 (91,051.6–163,778.2)			
Place of residence			0.252		
NSW metropolitan	204	107,146.4 (87,178.2–150,827.5)			
NSW regional or remote	162	114,588.3 (87,428.0–158,211.5)			
Interstate	93	107,193.8 (82,615.6–131,555.5)			
Overseas	2	181,449.0 (123,194.9–N/A)			
Neoadjuvant radiotherapy^b^			0.278		
Yes	207	107,250.1 (84,214.5–141,008.5)			
No	252	109,230.0 (88,011.6–152,974.6)			
Neoadjuvant chemotherapy^b^			0.527		
Yes	226	107,728.7 (84,246.9–142,837.6)			
No	234	108,726.9 (87,574.8–146,233.7)			
Type of PE			< 0.001	34,054.3 (7,007.9)	< 0.001
Complete	244	121,469.8 (101,789.6–163,491.9)			
Partial	217	92,771.8 (71,906.6–122,611.0)			
Tumour type^a^			0.500		
Rectal	283	108,259.4 (86,791.4–141,328.3)			
Other	160	113,318.8 (86,830.9–157,909.6)			
Operation number^a^			0.002		
Primary	207	103,433.6 (83,175.7–133,019.9)			
Recurrent	236	114,723.9 (92,305.4–161,620.6)			
Surgical intent			0.019		
Curative	439	107,777.1 (85,809.8–142,830.6)			
Palliative	21	142,788.9 (105,938.1–205,228.4)			
Combined CRS +/- HIPEC			< 0.001	76,448.1 (20,311.6)	< 0.001
Yes	14	195,521.5 (152,296.6–223,748.9)			
No	447	107,608.1 (86,006.9–141,328.3)			
Sacrectomy			0.038		
Yes	206	111,743.0 (92,041.5–146,790.1)			
No	255	105,135.9 (81,777.2–144,226.4)			
VRAM reconstruction			0.102		
Yes	41	119,348.1 (99,120.7–163,080.4)			
No	420	107,692.6 (84,504.2–142,851.5)			
Iliac vessel resection or repair^b^			< 0.001		
Yes	336	111,528.9 (90,776.1–152,146.6)			
No	124	100,301.6 (72,369.1–127,325.1)			
Sciatic nerve excision^b^			0.379		
Yes	73	118,607.8 (82,946.1–159,679.0)			
No	386	107,692.6 (86,889.5–142,837.6)			
Surgical margins^c^			0.534		
R0	369	108,675.7 (86,390.4–143,882.4)			
R1/2	77	109,409.6 (86,963.5–157,412.1)			

Data presented as median (IQR); All costs are 2022 $AUD

^a^PE for non-cancerous indications excluded

^b^Incomplete data

^c^15 cases were not assessable

^d^Model utilises continuous age data

Operations involving a vertical rectus abdominal flap (VRAM) reconstruction (*p* = 0.102) or sciatic nerve excision (*p* = 0.379) were not found to incur higher costs in the univariate analysis, whereas PE with iliac vessel resection or repair ($111,528.9 vs $100,301.6 AUD; *p* < 0.001) or with sacrectomy ($111,743.0 vs 105,135.9; *p* = 0.038) were associated with significantly higher total admission costs. The patients who experienced one or more postoperative complications also incurred significantly higher admission costs ($113,011.7 vs $74,308.5 AUD; *p* < 0.001). Neither year of surgery nor operative margins, defined as R0 versus R1/2, had a significant effect on total admission cost (*p* = 0.548 and 0.534, respectively) (Table 3).

Backward multivariate regression analysis identified advancing patient age, complete PE (vs partial PE), and PE in combination with CRS ± HIPEC to be significantly associated with increasing total admission cost of PE (Table 3). Specifically, the multivariate model found cost to increase by $770.4 AUD for each additional year of age (*p* = 0.006), by $34,054.3 AUD for a complete versus a partial PE (*p* < 0.001), and by $76,448.1 AUD if PE was combined with CRS ± HIPEC (*p* < 0.001; Table 3). The observed *R*^2^ was 0.08972, indicating that these factors combined accounted for less than 10 % of the total variation in cost. Other variables including patient sex, operation number, surgical intent, sacrectomy, and iliac vessel repair or reconstruction were not significant contributors to overall cost based on the backward multivariate analysis.

## Discussion

Despite increasing interest in PE surgery globally, the procedure remains relatively uncommon, even at specialist referral centers. Although reported survival and quality-of-life outcomes are favorable, such radical surgery may be associated with prolonged operating times, routine ICU admission, significant risk of major morbidity and postoperative complication, and substantial hospital LOS.^3,5,16–18^ Furthermore, funding for such low-volume, high-cost surgery is increasingly relevant in the context of rising health care costs and increasing attention on health expenditure.^8^ As such, this study provides valuable insight into the economic implications of PE and the most up-to-date account of hospital admission costs associated with PE at a specialist referral center.

Among 461 consecutive patients, the median total inpatient cost was $108,259.4 AUD (adjusted to 2022 $AUD prices). In 7 % of cases, total inpatient costs exceeded $250,000 AUD, with the highest reported cost in this study being $582,767.5 AUD. However, these cases are uncommon, with the majority of patients incurring total admission costs of between $86,620.8 and $144,429.3 AUD. The overall inpatient cost was positively skewed, likely reflecting the known risk of significant postoperative morbidity and extended hospital LOS associated with PE.^6,19^ This was corroborated by outliers accruing extreme costs, typically requiring multi-month postoperative admissions and multiple unplanned returns to the operating theater.

In this study, the costs incurred from staffing and the operating room were the major contributors to total admission costs. High operating room and staffing costs are anticipated given the often long and challenging intra- and postoperative course of patients undergoing PE. However, these findings reiterate the reliance on highly specialized multidisciplinary medical, nursing, and allied health staff for a successfully managed exenteration program. Expected prolonged operating times, ICU admission, and hospital stays, in addition to the substantial risk of complications, all undoubtedly contribute to the high total inpatient costs, which include proportional increases in staff and operating room costs.^5^ Regardless, these data demonstrate that staffing costs, whether in the operating room or on the ward during the postoperative period, contribute significantly to the overall cost of PE and, in turn, the importance of centralized services with adequate budgets to facilitate dedicated exenteration services.

The high cost of PE is driven by the highly complex nature of the procedure, with increasing surgical complexity associated with higher overall costs. Patients undergoing complete PE, PE for recurrent malignancy or with palliative intent, involvement of the iliac vasculature, sacrectomy, PE combined with CRS + HIPEC, and male patients all incurred significantly higher admission costs based on univariate analysis, likely reflecting the operative challenges associated with these cohorts. Interestingly, similar findings were not demonstrated for cases requiring VRAM reconstruction, but the lack of statistical significance may indicate type 2 error. Regardless, these results are notable given the overall surgical complexity of PE and the increasing rate of PE for recurrent malignancy.^20^

Multivariate analysis demonstrated that patient age, type of PE (curative vs palliative), and PE in combination with CRS ± HIPEC are significantly associated with overall cost. Notably, patient sex, operation number, surgical intent, sacrectomy, and iliac vessel resection or repair were found to be significantly associated with total cost in univariate but not multivariate analysis. Additionally, increasing patient age was not found to be significantly associated with cost in univariate analysis, but was found to be associated with significantly higher costs using backward multivariate regression. Together, these findings suggest that whereas confounding factors can be better accounted for with multivariate analyses, these variables alone contribute less than 10 % to the overall variance in cost. As such, factors not included in this model, possibly unexpected or un-anticipatable events, appear largely responsible for the overall cost variation between cases. This is corroborated by our finding that patients who experience one or more complications have a median cost more than 50 % greater than patients who do not experience any postoperative complication.

The published data detailing the costs associated with PE procedures in Australia and also globally are scarce. Data from the United Kingdom and Europe are lacking, but published data on the cost of PE from the United States are comparable with or higher than the costs demonstrated in this report. Miller et al.^21,22^ reported that the total inpatient costs associated with surgical resection of locally recurrent rectal cancer was $65,917 (U.S. dollars [USD]) in the year 2000 ($138,583 USD, adjusted for health care inflation to 2022 dollars). Althumairi et al.^22,23^ reported that the inpatient cost of PE for various pelvic tumors was $46,585 USD in 2016 ($55,156 USD, adjusted for health care inflation to 2022 dollars). Based on the exchange rate in July 2022, the median cost of PE based on the data from this report is $74,211.8 USD, exceeding that reported by Althumairi et al.,^22,23^ but notably less than that reported by Miller et al.^21,23^

Previous work by our group estimated the inpatient costs of PE to be $56,638 AUD, based on data from a patient cohort from 2008–2011.^7^ Accounting for health care-adjusted inflation, these costs equate to $70,473.8 in 2022 AUD, notably less than the median admission cost of $108,259.4 AUD incurred by the cohort described in this report.^8,14^ However, Koh et al.^7^ used on a “bottom-up costing” analysis for individual patients, in contrast to the “top-down costing” method of extracting iFRACS data used for this report. As such, differences in the total calculated costs may, in part, reflect the different methods used rather than significant differences in the true inpatient costs associated with PE. Specifically, the iFRACS data incorporate all nonclinical administrative costs as well as food and domestic services, patient transport, and accounting costs associated with the hospital, which may not be captured using a bottom-up costing method.

Importantly, however, data from the aforementioned studies were obtained from patients undergoing PE before 2011, and likely do not reflect the contemporary costs of inpatient surgical care.^7,21,23^ Substantial advances in surgical technique have been made during the past decade, with more complex resections such as high sacrectomy, pubic bone excision, and radical neurovascular excision currently performed routinely at specialized units.^5,20,24^

The authors have previously reported an increasing complexity of surgery over time at their unit, which, despite improved R0 rates and survival, has been associated with increased blood loss, morbidity, and hospital stay.^24^ It is likely that the total cost of a “routine” complete visceral exenteration (without bone or neurovascular resection) is substantially less than the costs reported in this, and that units in which these extended exenterative resections are not performed may expect lower costs. Interestingly, delivery of a PE service seems to cost less than the cytoreductive surgery program at the same institution, with a total inpatient cost of $130,804 AUD among a cohort of patients from April 2017 through June 2019.^11^

The strengths of this study included the large sample for what remains an uncommon procedure and the use of prospectively collected data allowing detailed and accurate costing analysis. The iFRACS data represent the actual costs incurred by the hospital rather than estimated costs based on indirect methods, allowing the true inpatient cost of admission to be reliably quantified.

However, this study did have some inherent limitations. Only inpatient costs were considered, with inability to report the overall treatment cost (including outpatient costs). This study also was largely descriptive and did not compare the cost of surgery with the cost of nonoperative or palliative management or attempt to perform a cost-benefit analysis. Additionally, this study was conducted at a high-volume specialist center, which performs a high proportion of complex bone and neurovascular resections, and therefore the findings may not be generalizable to units performing predominantly viscera-only resections.

Overall, the total admission cost for patients undergoing PE reflects the complexity of the surgical treatment requirement for multidisciplinary care. The costs associated with PE increase with advancing patient age, complete PE, and PE procedures combined with CRS ± HIPEC based on backward multivariate analysis. Strategies to reduce the cost of PE could focus on the implementation of routine prehabilitation programs to reduce rates of postoperative complications and postoperative LOS.^25^ However, further research is needed to define costs accrued from the pre- and post-admission periods, and robust cost benefit analyses are necessary to characterize the overall health economic impact of PE.

## Supplementary Information

Below is the link to the electronic supplementary material.

Supplementary file1 (DOCX 17 KB)
