# Supplementary material for: Cost Analysis of Pelvic Exenteration Surgery for Advanced Pelvic Malignancy
Source: Ann Surg Oncol. 2024 Sep 16;31(13):9079–87. doi: 10.1245/s10434-024-16227-3 (PMC11549131; doi:10.1245/s10434-024-16227-3)
Supplement: Supplementary file 1 — Supplementary file1 (DOCX 17 KB) [file 10434_2024_16227_MOESM1_ESM.docx]

| **Supplementary Table 1.** Detailed composition of each cost bucket | |
| --- | --- |
| **Cost bucket** | **Description** |
| **Critical care (ICU, HDU, CICU, NICU, CCU)** | - Goods & Services - Salary and Wages - VMO Payments for critical care cost centres |
| **Diagnostic** | - Pathology (blood and pathology goods & services) - Imaging (radiology goods & services) - Specialist procedure suites (goods & services, salary and wages and VMO payments for specialist procedures suites) - Pharmacology (pharmacy goods & services) - Prosthetics: (prosthesis costs) |
| **Emergency department** | - Goods & services - Salary and Wages - VMO Payments for emergency department cost centres |
| **Operating room** | - Goods & services - Salary and wages - VMO Payments for operating theatre cost centres |
| **Other** | - Hotel (food and domestic services) - Non-clinical (administrative costs, non-clinical salaries and wages) - On costs (superannuation and workers compensation premium payments) - Excluded (average costs that are excluded from the NSW State price including all depreciation, annual and long service leave, actuarial adjustment, interest) - Patient transport (patient transport costs in all cost centres) |
| **Staff** | - Medical (medical staff salary and wages, VMO payments in clinical service or ward cost centres) - Nursing (nursing salary and wages in clinical service or ward cost centres) - Allied Health (allied health staff salary and wages for allied health or clinical service cost centres) |
| **Ward** | - Goods & services for clinical service or ward cost centres. |
| CCU, coronary care unit; CICU, cardiothoracic intensive care unit; HDU, high dependency unit; ICU, intensive care unit; NICU, neurosurgical intensive care unit; VMO, visiting medical officer | |
